# New Generation Dielectrophoretic-Based Microfluidic Device for Multi-Type Cell Separation

**DOI:** 10.3390/bios13040418

**Published:** 2023-03-24

**Authors:** Pouya Sharbati, Abdolali K. Sadaghiani, Ali Koşar

**Affiliations:** 1 Faculty of Engineering and Natural Sciences, Sabanci University, Istanbul 34956, Turkey; 2 Sabanci University Nanotechnology and Applications Center (SUNUM), Sabanci University, Istanbul 34956, Turkey

**Keywords:** dielectrophoresis, lab-on-a-chip, 3D electrodes, blood cells, electrochemical cells

## Abstract

This study introduces a new generation of dielectrophoretic-based microfluidic device for the precise separation of multiple particle/cell types. The device features two sets of 3D electrodes, namely cylindrical and sidewall electrodes. The main channel of the device terminates with three outlets: one in the middle for particles that sense negative dielectrophoresis force and two others at the right and left sides for particles that sense positive dielectrophoresis force. To evaluate the device performance, we used red blood cells (RBCs), T-cells, U937-MC cells, and Clostridium difficile bacteria as our test subjects. Our results demonstrate that the proposed microfluidic device could accurately separate bioparticles in two steps, with sidewall electrodes of 200 µm proving optimal for efficient separation. Applying different voltages for each separation step, we found that the device performed most effectively at 6 Vp-p applied to the 3D electrodes, and at 20 Vp-p and 11 Vp-p applied to the sidewall electrodes for separating RBCs from bacteria and T-cells from U937-MC cells, respectively. Notably, the device’s maximum electric fields remained below the cell electroporation threshold, and we achieved a separation efficiency of 95.5% for multi-type particle separation. Our findings proved the device’s capacity for separating multiple particle types with high accuracy, without limitation for particle variety.

## 1. Introduction

Interpretation of the “electrical signature” of biological cells has been widely recognized during the last decades as a feasible approach in medical application [1]. Different external stimuli such as electrical field for electrophoresis [2,3] and dielectrophoresis (DEP) [4,5,6,7], acoustic wave for acoustophoresis [8], magnetic field for magnetophoresis [9,10], and light beam for photophoresis [11,12] have been used to identify and sort target cells under various experimental conditions. Among them, DEP has become popular in manipulating bioparticles [1,13,14]. DEP-based manipulation is easy to control by changing the electrical conductivity and permittivity of the suspending medium or the frequency and intensity of the applied electric field. Additionally, the use of alternating current (AC) minimizes electrolysis reactions of microelectrodes and Joule heating problems, which can damage cells [15]. As a result, DEP has been broadly applied on bioparticles such as DNA [16], RNA [17], proteins [18], bacteria [19], blood cells [20], and circulating tumor cells (CTCs) [21]. The electrical properties of cells can be measured using broadband dielectric spectroscopy (BDS) [22].

The DEP effect has been extensively used for separation [23,24], manipulation [25], and characterization [26] of the particles and biological cells. As an example, Wolff et al. utilized DEP as a method for single-molecular manipulation of DNA [27]. Honeyeh M. E. et al. used a DEP microfluidic device to characterize and separate live and dead yeast [28]. They presented a complementary metal-oxide semiconductor (CMOS)-integrated silicon-based microfluidic device with embedded arrays of integrated electrodes array. Their device is capable of rapid and selective cell separation. Kung et al. proposed a microfluidic device utilizing a long 3D Quadro-electrode to provide a tunable nonuniform electric field for size-based cell separation [29]. They obtained >90% purity with 1 µm particle size sorting resolution. In size-based cell separation, sorting resolution is crucial due to the small differences in size among bioparticles.

Although DEP is a commonly used method for cell separation, most DEP-based microfluidic devices have limitations in distinguishing and separating various types of particles within a single sample. For example, in a blood sample, different bioparticles coexist. However, most of existing DEP-based separation devices are only capable of dividing cells into two groups. As an example, Varmazyari et al. presented a microfluidic system to separate CTCs from different subtypes of white blood cells with high cell viability for postprocessing analysis [30]. Nguyen et al. proposed a microfluidic device based on DEP for cell separation and evaluated its performance through simulations. Their DEP microfluidic chip demonstrated an accuracy of 95% in separating three types of CTCs from red blood cells (RBCs) [31]. However, while their device demonstrated high accuracy in distinguishing RBCs from CTCs, it was unable to separate CTCs from each other. Other designs have been developed and were capable of separating multiple particles, such as Kazemi and colleagues’ DEP-based separation device optimized for size-based separation using the finite-element method [32]. Their design successfully separated three different types of particles, but it was unable to divide more than three groups of particles. Han et al. proposed a dielectrophoretic device utilizing sidewall electrodes for size-based separation [33]. Although their device was designed for continuous and simultaneous multi cell-types separation, it was limited to four groups and could not separate particles in a sample into more than four subgroups at the same time.

The microfluidic device proposed in this study is capable of separating multiple types of particles/cells from each other with no limitation in variety. The device utilizes two sets of electrodes to generate a nonuniform electric field within the microchannel: a set of 3D cylindrical electrodes at the beginning of the main channel to trap cells, and a set of 3D sidewall electrodes along the walls to lead cells to the desired outlets.

In the process of cell separation, maintaining cell viability is a critical concern for postprocessing analysis. Electric fields generated near the electrodes in a microfluidic device can reach intense levels, which can cause temporary disruption of the cell membrane when a cell enters such a region. This process is called electroporation and is used in several applications such as cancer treatment, drug delivery, and gene therapy. However, it is crucial to avoid cell damage during the separation process, as it may be irreversible and destroy the cell. Thus, it is important to minimize the applied electric field in DEP-based separation to ensure efficient separation without harming cells. The proposed microfluidic device in this study employs 3D cylindrical electrodes to provide better electric field coverage at a lower voltage, thereby decreasing the risk of cell damage.

To evaluate the performance of the device for cell separation, four bioparticles, namely RBCs, T-cells, U937-MCs, and Clostridium difficile bacteria, were selected, and the profile of the real part of Clausius–Mossotti (CM) factor for each particle, their cross-over frequency, and the profile of induced transmembrane voltage were obtained. The optimization of the 3D sidewall electrode size was conducted by evaluating three different sizes, i.e., 100 µm, 150 µm, and 200 µm, to determine the optimal size that achieves the highest accuracy while avoiding cell damage during the separation process. The electric field intensity applied to cells inside the microchannel was analyzed for similar operational parameters, and the results indicate that the 200 µm sidewall electrode size exhibited superior performance, with an electric field intensity below the electroporation threshold of the cells at the cells’ path.

Experimental investigations of cell separation processes are often challenging, time-consuming, and expensive. Therefore, modeling and simulation methods have been shown to be powerful tools for various cell manipulation applications in microfluidic chips [30,34,35]. Table 1 summarizes some of the recent studies on simulation-based analysis for this purpose. Computer simulations allow for the analysis and prediction of the electromechanical behavior of different cell types and their motion within the microfluidic devices. In this study, the performance of the proposed design was evaluated using theoretical calculations and numerical simulations. The validity of the method was confirmed by comparing the results with experimental results reported in the literature.

The proposed microfluidic design was able to successfully separate four bioparticles at a flow rate of 0.2 µL/min and a voltage of 6 Vp-p for 3D cylindrical electrodes. The sidewall electrodes’ voltage varied according to the size of the particles. For separating RBCs from bacteria, 20 Vp-p was applied, whereas 11 Vp-p was applied for separating T-cells from U937-MC. The results confirm the effectiveness of the proposed microfluidic design in separating multiple particles/cells without any limitation in variety, with minimal risk of cell damage.

## 2. Theory

### 2.1. Dielectrophoresis

In dielectrophoresis, a particle suspended in a fluid medium is placed in a nonuniform electric field. The electric field polarizes the particle so that the DEP force is generated. The DEP force acting on a spherical particle can be obtained as [39,40]:

(1) $$F_{DEP}=2\pi \varepsilon_{m}r^{3}Re\left[f_{CM}(\omega )\right]\nabla \left|E_{rms}^{2}\right|,$$

where $\varepsilon_{m}$ is the absolute permittivity ($\varepsilon_{0}\varepsilon_{r})$ of the medium surrounding the particle, $\varepsilon_{r}$ is the relative permittivity of the medium, $\varepsilon_{0}=8.85\times 10^{-12}Fm^{-1}$, r is the radius of the particle, $Re\left[f_{CM}(\omega )\right]$ is the real part of Clausius–Mossotti factor, ω is the frequency of the applied electric field, and $\nabla |E_{rms}^{2}|$ is the gradient of the electric field. The Clausius–Mossotti factor describes the relative polarizability of the particles and the surrounding medium:

(2) $$f_{CM}(\omega )=\frac{\varepsilon_{eff}^{*}-\varepsilon_{m}^{*}}{\varepsilon_{eff}^{*}+2\varepsilon_{m}^{*}},$$

(3) $$\varepsilon_{eff}^{*}=\varepsilon_{mem}^{*}\frac{{\left(\frac{r}{r-d}\right)}^{3}+2\frac{\varepsilon_{int}^{*}-\varepsilon_{mem}^{*}}{\varepsilon_{int}^{*}+2\varepsilon_{mem}^{*}}}{{\left(\frac{r}{r-d}\right)}^{3}-\frac{\varepsilon_{int}^{*}-\varepsilon_{mem}^{*}}{\varepsilon_{int}^{*}+2\varepsilon_{mem}^{*}}},$$

(4) $$\varepsilon^{*}=\varepsilon -j\frac{\sigma }{\omega },$$

where $\varepsilon_{m}^{*}$ and $\varepsilon_{eff}^{*}$ are the complex permittivity of the medium and the effective complex permittivity of the cell, respectively [41]; $\varepsilon_{int}^{*}$ and $\varepsilon_{mem}^{*}$ are the mixed permittivity of the cytoplasm and membrane, respectively; d is the thickness of the membrane; ε is the absolute permittivity; σ is the conductivity; ω is the angular frequency of the applied electric field; and $j=\sqrt{-1}$. If the real part of the CM factor is positive, particles experience a positive DEP force (pDEP) and are attracted to the areas of high electric field gradients. In the case of a negative value of the real part of the CM factor, particles are exposed to a negative DEP force (nDEP), are repelled from the regions with a high gradient of electric field, and move toward the areas with a weak electric field gradient.

### 2.2. Electroporation

The effect of exposing a cell to an electric field is the generation of voltage in the cell membrane, which is called induced transmembrane voltage (*V_c_*). When a sufficiently strong electric field is applied to a cell, the resulting transmembrane voltage can surpass the physiological range of the cell. This might lead to nonphysiological consequences, including the rearrangement of lipids in the membrane bilayer, which forms and stabilizes pores, altering the cell’s dielectric properties (electroporation). The steady state of the transmembrane voltage generated in cells with sinusoidal electric fields is analytically expressed as [42,43]:

(5) $$V_{c}=f_{c}ERcos\theta \frac{1+j\omega \tau_{m2}}{1+j\omega \tau_{m1}},$$

(6) $$f_{c}=\frac{3\sigma_{e}[3\delta R^{2}\sigma_{cyt}+(3d^{2}R-d^{3})(\sigma_{mem}-\sigma_{cyt})]}{2[R^{3}\left(\sigma_{mem}+2\sigma_{e}\right)\left(\sigma_{mem}+\frac{1}{2}\sigma_{cyt}\right)-{\left(R-\delta \right)}^{3}(\sigma_{e}-\sigma_{mem})(\sigma_{cyt}-\sigma_{mem})]},$$

(7) $$\tau_{m1}=\frac{\varepsilon_{mem}}{\frac{d}{R}\times \frac{2\sigma_{cyt}\sigma_{e}}{\sigma_{cyt}+\sigma_{e}}+\sigma_{mem}},$$

(8) $$\tau_{m2}=\frac{\varepsilon_{cyt}+{2\varepsilon }_{e}}{\sigma_{cyt}+2\sigma_{e}},$$

where *R* is the cell radius, *E* is the electric field intensity, *τ_m_*_1_ and *τ_m_*_2_ are the membrane’s first and second time constant for describing the frequency dependence of *V_c_*, respectively, and *θ* is the angle of the given membrane position and electric field direction. *σ_cyt_*, *σ_mem_*, and *σ_e_* are the conductivities of the cytoplasm, cell membrane, and external medium, respectively, and *d* is the membrane thickness.

Electroporation can be either reversible or irreversible, depending on the extent of structural changes in the membrane. When the electric field is removed, the pores in the reversible case heal, and the bilayer is resealed. In the irreversible case, however, the pores expand excessively, causing the mechanical rupture of the cell membrane.

## 3. Methodology

### 3.1. The Microfluidic Device

**Figure 1:**
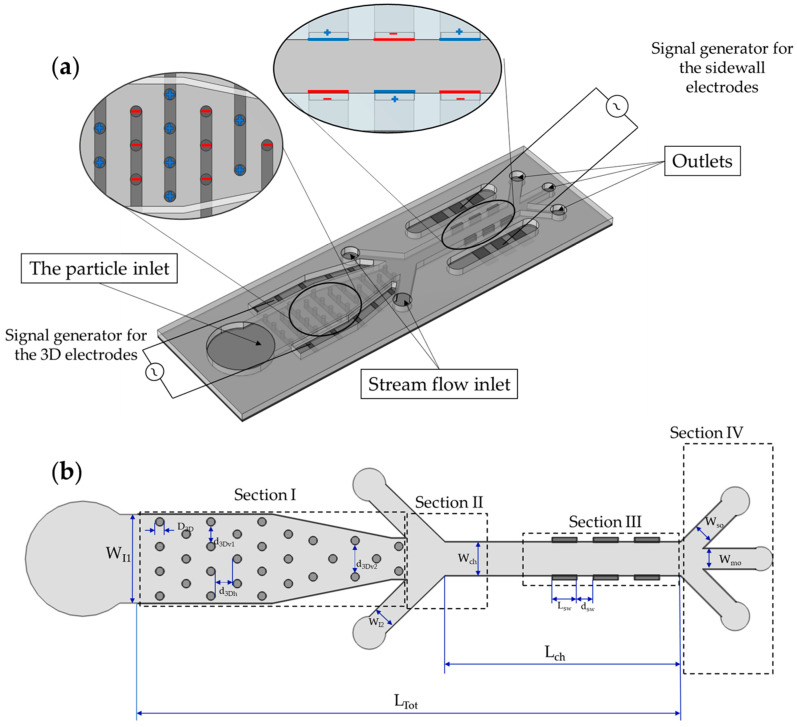
(**a**) Schematic of the microfluidic device; (**b**) Top view of the microfluidic channel with different sections.

The schematic of the microfluidic device is shown in Figure 1a. There are two types of electrodes in the device: (i) the 3D cylindrical electrodes in Section I (Figure 1b), and (ii) the 3D sidewall electrodes placed within the walls of the microchannel in Section III (Figure 1b). In this study, 3D cylindrical electrodes will be referred to as “3D electrodes”, while 3D sidewall electrodes will be referred to as “sidewall electrodes”. For each electrode set, there is a separate signal generator, which allows the voltage and frequency to be independently adjusted. The device contains twenty-nine cylindrical electrodes, which are located in Section I (Figure 1b), the initial place for cells to go after entering the device. The sidewall electrodes are located in Section III, close to the outlets (Figure 1b). Three lengths (L_sw_) of 100 µm, 150 µm, and 200 µm were considered in order to determine the optimal size of sidewall electrodes. The fluid flows from these inlets, and the particles focus on the middle of the channel so that they will experience the same DEP force from the sidewall electrodes. The dimensions of the design are tabulated in Table 2.

### 3.2. Working Principle of the Microfluidic Device

**Figure 2:**
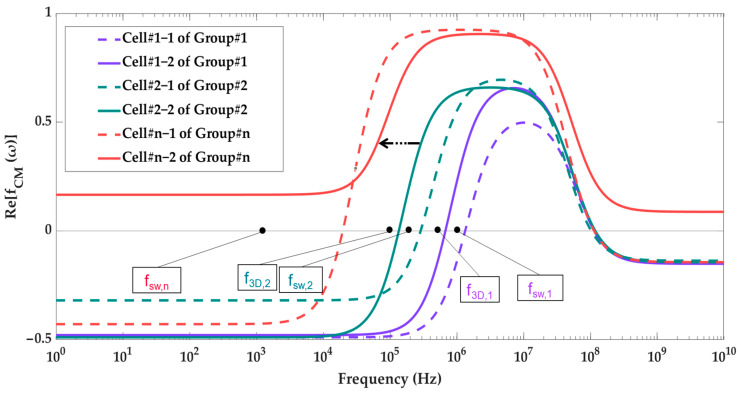
The real part of Clausius–Mossotti (CM) factor for cells of group 1 to n.

The working steps of the device are shown in Table 3. Accordingly, there are 2 × *n* types of cells that need to be separated. Firstly, the microchannel is sterilized and is filled with buffer, ensuring that there are no bubbles present. Next, the sample is inserted into a tube connected to the sample inlet, while the buffer tube is connected to that tube. The cells are then propelled into the device through the flow of buffer so that all cells can be introduced as a single sample. They are divided into n groups, each of which contains two types of cells. Grouping is based on the real part of the CM factor. In order to accomplish this, we begin with the highest first crossover frequency. The two cells that have the highest first crossover frequency will be the cells in Group number one (Figure 2).

Group number two has the cells whose crossover frequencies are right after Group one cells (Figure 2). This procedure continues until the last two cell types.

The cells enter the separation device from the particle inlet (Figure 1a) and move to Section I. The frequency of the 3D electrodes is set to f_3D,1_ (Figure 2), at which all the cells are trapped by 3D electrodes except cell types in Group 1. They pass through Section I and are focused on the middle of the channel by the flow from extra inlets in Section II.

Upon arriving at Section III, they experience DEP force from the sidewall electrodes whose electric field frequency is f_sw,1_. As can be seen in Figure 2, cell number 1-1 experiences nDEP and exits from the middle outlet, while cell number 1-2 experiences pDEP and exits from the side outlet (Table 3, Step I). The first step is thus complete, and two cells are separated. Step II starts with the change in the frequency of the 3D electrodes’ electric field to f_3D,2_ so that cells of Group two will be repelled from the 3D electrodes and leave Section I toward the outlets. In this step, the frequency of sidewall electrodes is set to f_sw,2_ so that cell 2-2 is attracted, and cell 2-1 is repelled. Thus, the cells could be separated (Table 3). By adjusting the electrical parameters of the AC generators, these steps continue until the last group of cells which corresponds to Group n. When all the other cells are separated from each other except Group n, the signal generator of 3D electrodes will be turned off so that the cells will be released from the electrodes, and the flow guides them to Section III where the frequency of sidewall electrodes is set to f_sw,n_. Thus, cell n-1 exits from the middle outlet, and cell n-2 leaves through the side outlets.

It can be noted that the voltage of the applied electric field in each step depends on the cell size and flow rate, which we will discuss in this study. The number of required 3D electrodes is contingent upon the number of cells that need to be trapped. As a result, each design has a maximum capacity for the number of cells it can handle.

To ensure optimal device performance, the voltage applied to the 3D electrodes should be strong enough to keep the trapped cells on each electrode. If the voltage is too low, the drag force and cell-to-cell collisions will cause trapped cells to be removed. On the other hand, if the voltage is too high, the DEP force can become so strong that it traps a large number of cells on each electrode, resulting in channel blockage and decreased device performance.

### 3.3. Governing Equations

#### 3.3.1. Electric Field

An AC voltage is applied to the electrodes to have a nonuniform electric field inside the microfluidic channel. The electric field distribution inside the channel is modelled using the Laplace equation [44]:

(9) $$\nabla .J=Q_{i},$$

(10) $$J=\sigma E+J_{e},$$

(11) E=−∇φ,

where J is the current density, $Q_{i}$ is the charge density, σ is the electrical conductivity, E is the electric field, $J_{e}$ is the external current density, and φ is the applied potential. To have ${2V}_{0}V_{p-p}$ applied voltage, ${-V}_{0}$ is applied on negative electrodes, and ${+V}_{0}$ is applied on positive electrodes. The boundary condition of other parts of the channel is expressed as:

(12) n·J=0,

This boundary condition implies that the wall surfaces have electrical insulation. AC voltages with the 180° phase difference are applied to the electrodes. Solving these equations enables us to obtain the distribution of the electrical field gradient and to calculate the DEP force.

#### 3.3.2. Creeping Flow

Reynolds number is well below unity, and the medium is incompressible inside the channel. Incompressible creeping flow is considered to model the fluid flow. The flow field is governed by the continuity and Navier–Stokes equations as follows [45]:

(13) $$\nabla \cdot \vec{u}=0,$$

(14) $$\rho \left[\frac{\partial \vec{u}}{\partial t}+(\nabla .\vec{u})\vec{u}\right]=-\nabla \vec{p}+\mu \nabla^{2}\vec{u},$$

where $\vec{u}$ is the velocity vector, t is the time, ρ is the density of the solution, μ is the viscosity of the solution, and ∇p is the pressure gradient. The inlet boundary condition is expressed as $\vec{u}=100\;\mu m/s$, and the outlet boundary condition is imposed as P = P_0_. No slip boundary condition is considered for the walls.

#### 3.3.3. Particle Tracing

The main forces acting on the cells are the dielectrophoretic force (F_DEP_) and hydrodynamic drag force (F_Drag_). The gravity and other forces such as the virtual mass force and basset force are neglected due to the small size of the cells and large electrical field intensity [30,46]. The magnitude of DEP force on red blood cells is in the scale of pN (10^−12^ N), while the gravity force on an RBC is in the scale of 10^−14^ N, which is 100 times smaller. As a result, the omission of these forces will not affect the results significantly.

The movement of cells is governed by Newton’s second law [47]:

(15) $$m_{p}\frac{d(v_{p})}{dt}=F_{DEP}+F_{Drag},$$

(16) $$F_{Drag}=6\pi \eta r(\vec{u}-\vec{v_{p}}),$$

where η is the dynamic viscosity of the medium, $\vec{u}$ is the fluid velocity, $\vec{v_{p}}$ is the cell velocity, and r is radius of cell. F_DEP_ is obtained from Equation (1).

### 3.4. Numerical Modeling

#### 3.4.1. Simulation

**Figure 3:**
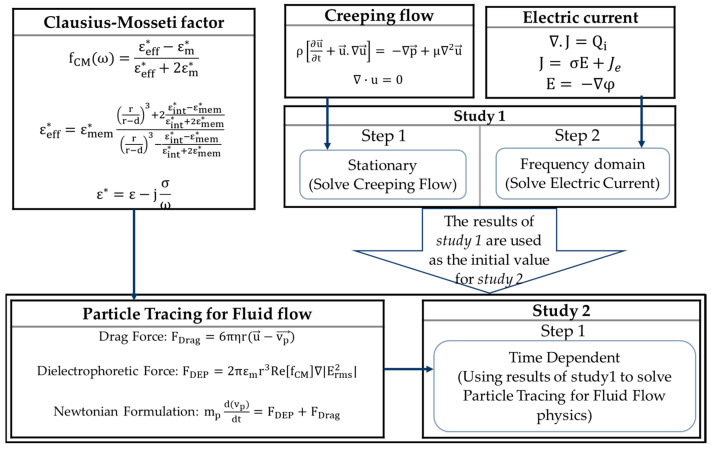
Flowchart with governing equations.

A numerical model was developed using the software COMSOL Multiphysics 5.6 and MATLAB R2020b [48] to solve the governing equations and to obtain the motion trajectory of cells. For this purpose, Creeping Flow (spf), Electric Currents (ec), and Particle Tracing for Fluid Flow (fpt) physics were used. Figure 3 shows the COMSOL simulation flow chart including governing equations and solver of each module. As the device is homogeneous in the z-direction and the electric field, and the electric field gradient remains constant in this direction, the simulations were performed in 2D. The first step of the Finite Element analyses started with obtaining the electric field distribution and fluid flow characteristics inside the microfluidics device. Here, the frequency-domain and stationary studies were used in the electric current and creeping flow modules, respectively. The results of this step were utilized as the initial value for step two (time-dependent analysis) for finding of the particle trajectory in the numerical domain. The electric field distribution from the frequency-domain study was helpful in the calculation of the DEP force in the transient study (Equation (1)), while the fluid flow velocity from the stationary study was used to calculate the drag force (Equation (16)). Equations (2)–(4) were numerically solved using MATLAB (R2020b), and the profile of the real part of Clausius–Mossotti factor was plotted for each particle. These profiles provided the crossover frequencies for each cell. The pDEP and nDEP frequency intervals for each cell could be determined based on these crossover frequencies.

To investigate the performance of the proposed DEP microfluidic device in this study, four types of bioparticles, namely red blood cells (RBC), T-cells, U937-MC, and Clostridium difficile bacteria (Bacteria), were employed. Table 4 displays their properties. The properties of the suspending solution are stated as: density = 1050 [kg/m^3^], permittivity = 80, and conductivity = 0.01 [S/m].

The device performance was investigated with three lengths of sidewall electrodes and different applied voltages to determine the optimum conditions for each step of separation.

#### 3.4.2. Validation and Mesh Independency

**Figure 4:**
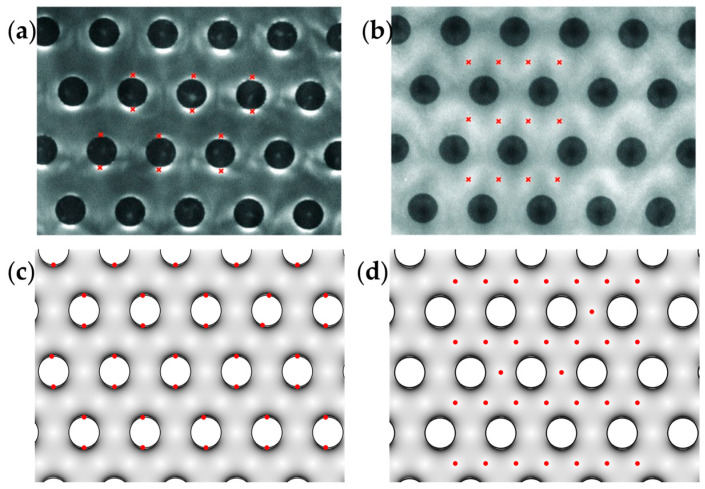
The particle spatial locations for positive dielectrophoresis force (pDEP) (**a**,**c**) and positive dielectrophoresis force (nDEP) (**b**,**d**). (**a**) Concentration of DNA on the edges of 3D electrodes in pDEP [50]; (**b**) Concentration of DNA in the distances between 3D electrodes in nDEP [50]; (**c**) The result of validation simulation in pDEP that shows particles stuck to the edge of 3D electrodes; (**d**) The result of validation simulation in nDEP that shows particles located at the gaps between the electrodes.

**Figure 5:**
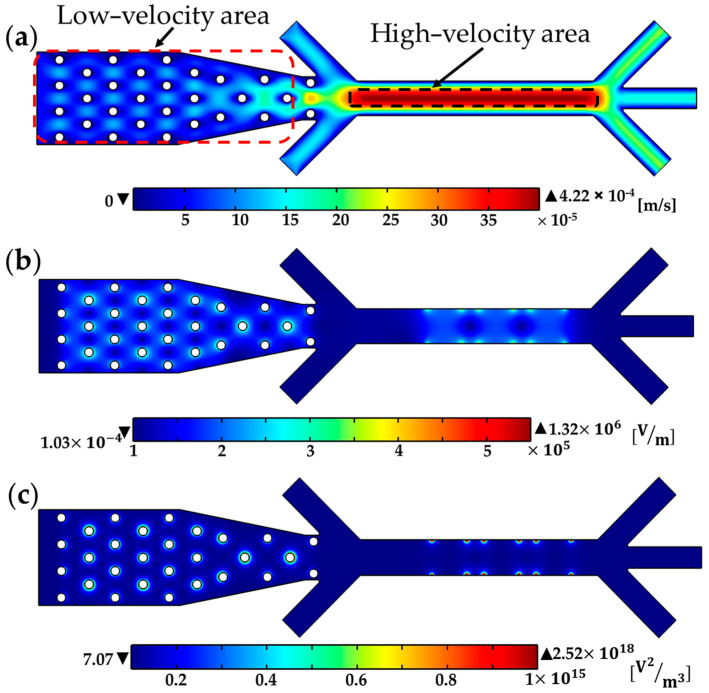
(**a**) Velocity field in the microchannel; (**b**) Electric field distribution in the microchannel; (**c**) Electric field gradient distribution in the microchannel.

The numerical model was validated against the experimental data of the study conducted by Martinez et al. [50]. The electric field gradient distribution and the particle concentration under the nDEP and pDEP conditions of the developed model were compared with the experienced ones in the literature [50]. Figure 4a,b shows the exemplary fluorescence microscopy images from the Martinez et al. [50] study displaying DNA concentration in the carbon-electrode array. The red crosses in Figure 4a and Figure 5b indicate the areas where DNA is concentrated. Figure 4a shows that DNAs gather around the 3D electrodes in pDEP. Figure 4c is the result of the simulation, which clearly shows that the cells in pDEP around the 3D electrodes concentrate in high electric field gradient areas. Figure 4b illustrates that DNAs have been repelled from the edges of electrodes due to nDEP, and their intensity is larger in the areas between electrodes. Figure 4d displays the location of particles in nDEP, which are in the regions with a low gradient of the electric field between electrodes. As can be seen, a good agreement between the simulation results and experimental data exists.

For the finite element analysis, the microchannel should be discretized using triangular meshes of different sizes. Finer meshes were assigned to the areas around electrodes due to the expectation of larger electrical field gradients. Therefore, meshing with physics-controlled elements was chosen. For mesh independency studies, the electric field and electric field gradient in the microchannel were considered to save computation time in the generation simulation results. Five different mesh sizes of extremely fine, extra fine, finer, fine, and normal were considered. Based on the simulations conducted with these mesh sizes, the electric field and gradient of the electric field distribution obtained from extremely fine and extra fine meshes differed by only 0% and less than 0.5%, respectively. Thus, the normal mesh was used for the computational results presented in this study. Therefore, the presented computational results are based on extra fine meshing with maximum element size of 47.6 µm and minimum element size of 0.179 µm.

## 4. Results and Discussion

### 4.1. Numerical Modeling

The fluid flow in the microfluidic channel is depicted in Figure 5a, for an inlet flow rate of 0.2 μL/min and the buffer inlet flow rates of 0.1 μL/min. The low flow rate in Section I allows for efficient cell attraction by 3D electrodes at lower voltages, reducing the potential for Joule heating and high voltage-induced cell damage. In contrast, the high velocity field in Section III prevents cell entrapment by sidewall electrodes and instead only directs cells towards their desired outlets by DEP force, in accordance with the sign of Re[f_CM_(ω)].

The electric field distribution is shown in Figure 5b. The uniformity of the electrodes in the z-direction results in a constant electric field in that direction. The effective coverage of the electric field around the cylindrical electrodes (Section I) makes it possible for all particles to experience the DEP force, allowing for easy trapping. Despite the decrease in electric field intensity in Section III due to the wider channel, the electric field from the sidewall electrodes still extends across the entire width of the channel.

Figure 5c depicts the gradient of the electric field distribution. The regions with strong electric field gradients (indicated by dark red color in the figure) attract particles that undergo pDEP. Conversely, the regions of electrode edges, which display strong electric field gradients, repel particles that undergo nDEP. As a consequence, during the trapping step, cells undergoing pDEP adhere to the edges of the 3D electrodes, whereas cells undergoing nDEP pass through Section I via the spaces between electrodes.

**Figure 6:**
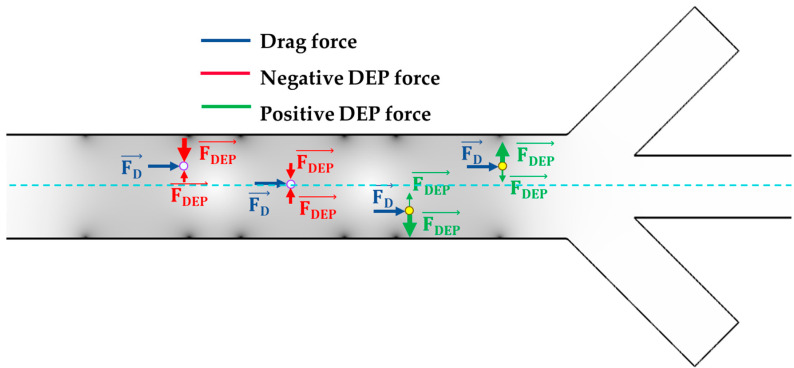
The applied forces on particles at different y positions.

Figure 6 illustrates the forces acting on particles in both pDEP and nDEP at different y-positions within Section III. As shown in the figure, the closer a particle is to the electrodes, the stronger the DEP force it experiences due to the increased intensity of the electric field. In the nDEP scenario, the stronger negative force from the nDEP dominates and propels the particle towards the center of the channel, until it reaches a y-position where the DEP forces from both sides are balanced. At this point, the drag force pushes it towards Section IV, and if it so chooses, towards the middle outlet. As a result, the particle in nDEP experiences a high DEP force as it enters to Section III, which then decreases as it moves further from the sidewall electrodes towards the center of the channel. In the pDEP scenario, the particles are attracted to the electrodes. As they move closer to Section IV, they experience an increasingly stronger DEP force due to their proximity to the higher intensity of the electric field. The drag force in Section III solely pushes the particles towards Section IV, while the DEP force guides them to their intended outlets (the side outlets for pDEP particles and the middle outlet for nDEP particles).

**Figure 7:**
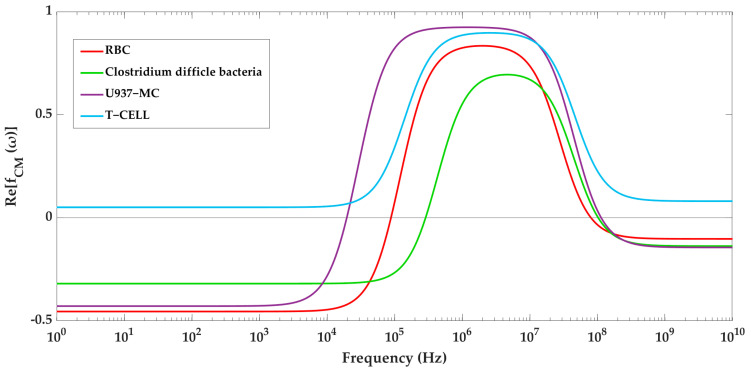
The real part of CM factor for the bioparticles.

It is crucial to determine the right frequency of the applied voltage to achieve an effective separation. Figure 7 displays the profile of the real part of CM factor over frequency for each cell type, which helps to determine the DEP behavior of particles. The graphs are constructed based on Table 4 and Equations (2)–(4). The crossover frequencies, at which Re[f_CM_(ω)] = 0, can be identified from this profile. As a result, the range of frequencies that result in either pDEP or nDEP can be obtained. The T-cells never experience nDEP and are always attracted to regions of strong electric fields, regardless of the frequency applied. The U937-MC cells have two crossover frequencies (19.4 kHz and 102 MHz), experiencing pDEP when the frequency of the applied electric field falls between these two frequencies, and nDEP outside of this range. The crossover frequencies for RBCs are 92 kHz and 77 MHz, and for bacteria cells, they are 290 kHz and 100 MHz.

These values were used for grouping cells into two groups and adjusting the signal generators for particle separation. Group 1 includes RBCs and bacteria, while T-cells and U937-MC belong to Group 2. According to Figure 7, the 3D electrode signal generator frequency should be set to values between 19.5 kHz and 92 kHz so that the 3D electrodes can capture T-cells and U937-MC during the initial stage of the separation process. Meanwhile, RBCs and bacteria continue with their journey towards Section III. Within the range of 92 kHz to 290 kHz, RBCs experience pDEP, while bacteria experience nDEP. However, to separate RBCs from bacteria using this frequency range, high voltage levels are needed to produce enough DEP force on small particles. High electric field intensity has a negative impact on cell viability. To overcome this challenge, a higher frequency can be used where both cell types are subjected to pDEP. By adjusting the voltage appropriately, RBCs, which are larger and have a higher Re[f_CM_(ω)] than bacteria, will experience a stronger DEP force and will be pulled towards the channel wall, while the force on bacteria will not be enough to divert it from its path towards the middle outlet. At 1.1 MHz, RBC’s Re[f_CM_(ω)] is close to its maximum so that a lower electric field intensity is sufficient to attract them. Meanwhile, bacteria have a 33% lower Re[f_CM_(ω)] and a smaller size ((5.6)^3^ times smaller), resulting in 233 (1.33 × (5.6)^3^) times weaker pDEP force compared to RBCs (Equation (1)). Therefore, we set the sidewall electrode frequency at 1.1 MHz to separate RBCs from bacteria. The second step of separation begins by turning off the voltage of the 3D electrodes, freeing T-cells and U937-MC, and the drag force directs them to Section III. By using a voltage frequency of 10 kHz on the sidewall electrodes and applying pDEP on T-cells and nDEP on U937-MC, T-cells will exit through the side outlets, while U937-MC will be guided to the middle channel.

### 4.2. Cell-Throuput Optimization of the Microfluidic Device

To this end, a mixture of red blood cells, T-cells, U937-MC, and Clostridium difficile bacteria is delivered into the suggested device, and their trajectories are captured in the presence of an applied electric field. Numerous simulations were performed for 3D electrodes and the three sidewall electrode sizes, 100 µm, 150 µm, and 200 µm, to evaluate the optimal performance of the system. Different voltages and flow rates were considered in each case, and the minimum voltage for effective trapping and separation was selected.

The voltage and frequency of the applied electric field from each electrode can be adjusted to have high accuracy from the device. The device should be operated according to particle size and the real part of CM factor profiles. In step I, f_3D_ = 35 kHz and f_sw_ = 1.1 MHz, and in step II, f_3D_ = off and f_sw_ = 10 kHz, where f_3D_ represents the frequency of the electric fields of 3D electrodes, and f_sw_ represents the applied frequency on sidewall electrodes.

The operation setting for 3D electrodes is V = 6 V_p-p_ at 35 kHz. This setting will generate a sufficient DEP force, capable of trapping cells and preventing their removal by drag force. Furthermore, the low voltage employed in this setting will prevent blockage of the channel, even if more than two layers of cells become trapped on the electrodes, by enabling the drag force and cell collisions to displace the extra cells. The good electric field coverage in Section I ensures that cells are trapped at the first available electrode, maximizing electrode utilization and preserving availability for future cell trapping.

**Figure 8:**
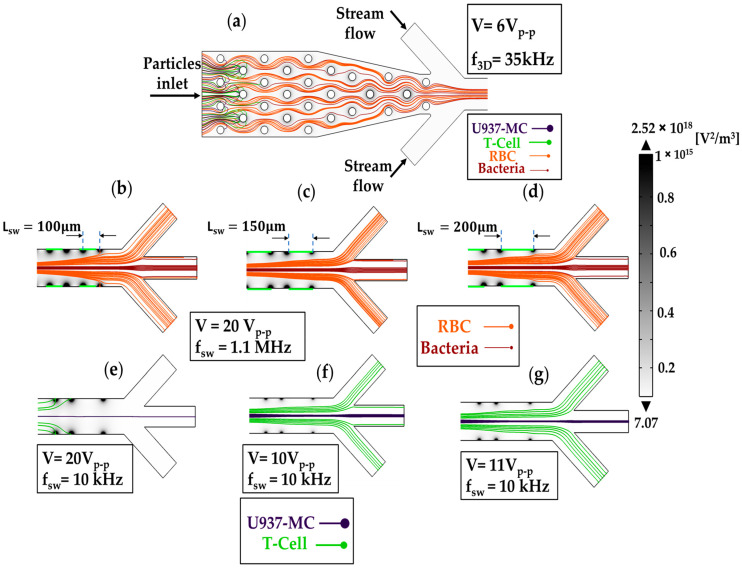
Cell trajectory in different steps of separation in Section III. (**a**) The cells of Group 1 are trapped by the 3D electrodes, and cells of Group 2 pass Section I. (**b**–**d**) Separation of cells in Group 1 by different sidewall electrode sizes. (**e**–**g**) Separation of cells in Group 2 under different applied voltages.

Figure 8a illustrates the cell trajectories in Section I and Section II. T-cells and U937-MC cells are found at the edge of 3D electrodes, where the electric field gradient is the highest. In contrast, RBCs and bacteria cells move between electrodes and pass through Section I. In Section II, the stream from the buffer inlets aligns cells in the middle of the channel, guiding them towards Section III. The cell trajectories of Group 1 in Section III for various sizes of sidewall electrodes are presented in Figure 8b–d, and the trajectories of T-cells and U937-MC for three different applied voltages in Section III are depicted in Figure 8e–g.

To evaluate the performance of the microfluidic device, three metrics are used: recovery rate, selectivity, and separation efficiency. The recovery rate measures how many cells of a certain type exit through the desired outlet compared to the total number entering the device. The selectivity measures the ratio of the desired cells in a target outlet to the total number of cells in that outlet. The separation efficiency measures the ratio of the desired cells that reach the target outlet to the total number of those cells separated across all outlets.

During the simulation, 500 RBC/s, 10 bacteria/s, 20 T-cell/s, and 5 U937-MC/s entered into the device, with the initial cell positions selected randomly. The entire mixture of cells entered the microchannel within 120 s, and the last cell of group 1 was extracted from the outlets at the 156th second. The trapped cells were then released and separated within 32.5 s. Many simulations were performed, and their results exhibited an outstanding performance, with the recovery rate for RBCs, bacteria cells, T-cells, and U937-MC cells obtained as 98.2%, 95.5%, 99%, and 100%, respectively. The simulations were repeated several times to ensure accuracy in the recovery rate calculation, and the average of these values was reported as the final recovery rate.

According to the simulation results, the average selectivity and separation efficiency of the proposed system were found to be approximately 99.9% and 99.8% for RBCs, 92.5% and 95.5% for bacteria, 100% and 98.9% for T-cells, and 95.8% and 100% for U937-MC.

After separating the cells, the viability of the isolated cells was evaluated. As previously mentioned, an alternating electric field (AC) was utilized to reduce the occurrence of electrolysis reactions of microelectrodes and to minimize Joule heating problems. The dielectrophoresis (DEP) method relies on the application of high electric fields to separate cells so that it is necessary to investigate the possibility of cell electroporation inside the microchannel. In this regard, the transmembrane voltage applied to the cells near the electrodes should not exceed a critical value.

**Figure 9:**
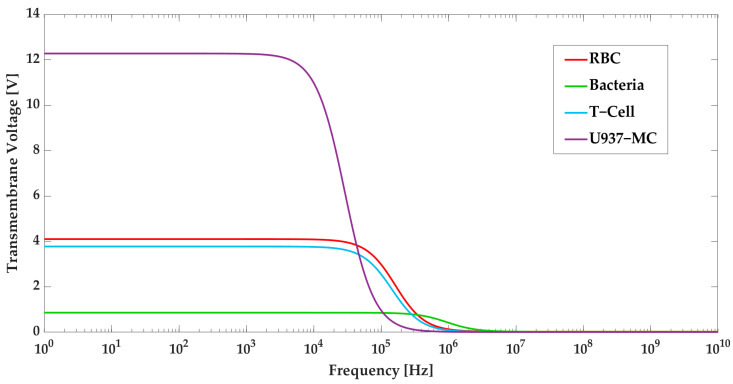
Transmembrane voltage for U937-MC, T-cells, RBCs, and bacteria as a function of frequency.

Based on Equations (5)–(8) and the data presented in Table 4, the relationship between the transmembrane voltage and frequency for each cell type is illustrated in Figure 9. As can be seen, at 35 kHz, the critical transmembrane voltage for RBCs, bacteria, T-cells, and U937-MC are 3.9 V, 0.86 V, 3.56 V, and 5 V, respectively. Meanwhile, at 1.1 MHz, the critical transmembrane voltages for RBCs and bacteria are 0.11 V and 0.375 V, respectively, and at 10 kHz, the critical transmembrane voltages for T-cells and U937-MC are 3.7 V and 10.8 V, respectively.

**Figure 10:**
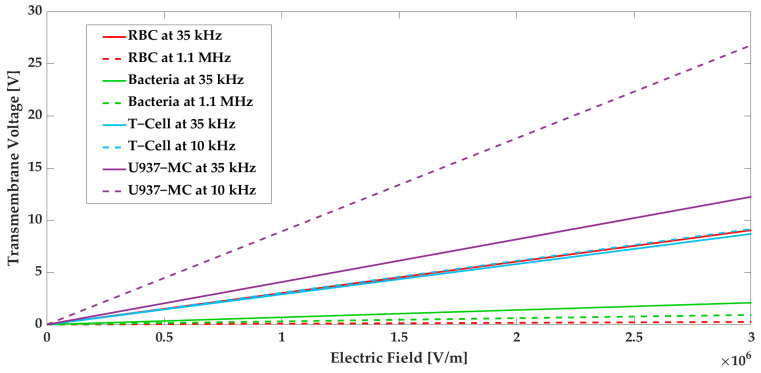
Transmembrane voltage for U937-MC, T-cells, RBCs, and bacteria as a function of electric field.

In order to maintain the viability of the cells, the induced transmembrane voltage should be less than the aforementioned values at each step. The transmembrane voltage is investigated for the various electric fields to obtain the maximum acceptable electric field for cell viability. Figure 10 displays the transmembrane voltage for each cell type as a function of the electric field. According to simulation results and Figure 10, the applied electric field in Section I is below the critical value. In Section III, the applied frequency for each group of cells is different. Therefore, the graph is plotted accordingly. In Section III, although the maximum electric field exceeds the acceptable limit for RBCs, the high electric field is only present at the edges of the sidewall electrodes (which is not on the RBCs’ path) and thus does not affect the RBCs’ viability. The electric field intensity in the path of the cells is well below the critical value. For the other cell types, their viability is preserved since they are exposed to electric fields that do not exceed the critical values.

**Figure 11:**
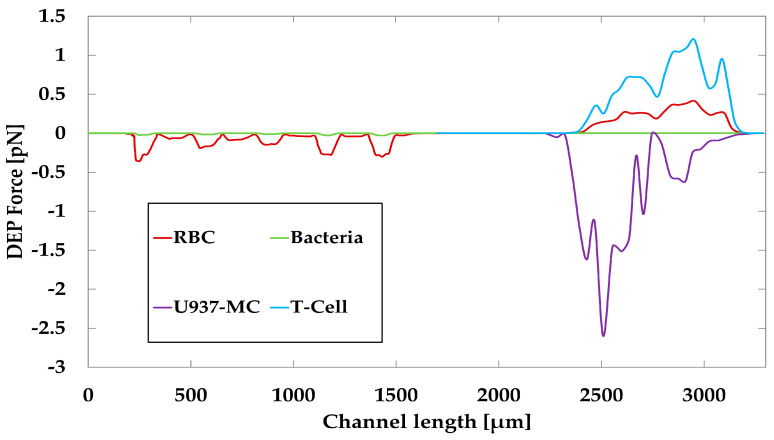
Applied doelectrophoresis (DEP) force on cells along the microchannel.

Figure 11 shows the applied DEP force on each particle along the microchannel. In the first separation step, T-cells and U937-MC cells were trapped. Therefore, their profile displays only the applied DEP force in Section III. The maximum forces acting on U937-MC cells, T-cells, RBCs, and bacteria are 872 pN, 84 pN, −0.41 pN, and −0.028 pN, respectively. There are two reasons to explain the large difference in maximum forces. First, T-cells and U937-MC cells stick to the electrode edges that have the highest electric field gradients, whereas RBCs and bacteria cells are repelled from those areas. The second reason is their size. The diameter of U937-MC is more than twice that of the T-cell, and the particle size has a power of three in the equation of DEP force (Equation (1)). The DEP force applied to trapped cells prevents their removal by drag force and cell collision. The drag force on the cells is weaker by an order of magnitude as compared to the DEP force that trapped them. Figure 11 also implies that the DEP force changes several times from maximum to minimum, which is caused by the nonuniformity of the electric field along the channel. It can be also seen that a trend in the value of the maximum in the profile of particles in pDEP in Section III is increasing, and the absolute value of the minimum in the profile of particles in nDEP in Section III has a decreasing trend. The reason for this result is the y location of the particles (Figure 6).

The proposed design can separate multiple types of bioparticles with high accuracy by using 3D cylindrical electrodes with 3D sidewall electrodes. As mentioned above, the particle size plays an important role in DEP-based microfluidic devices, and the particles in this study were selected in a wide range of sizes (1–14 µm in diameter) to reveal the potential of the device in cell separation. Emmerich et al. [51] proposed a microfluidic device to separate particles based on their size, but their design cannot separate particles whose sizes are equal or close to each other while our design can separate cells based on their size or their electrical polarizability.

While many studies use sidewall electrodes [33,52] or 3D electrodes [30,53] on the side walls of the microchannel to separate multiple bioparticles, most of them can only separate particles into two groups. Moreover, there are studies that utilized microfluidic cell separation devices using only 3D electrodes [54], whose designs were not capable in multicell-type separation. A device with 3D cylindrical electrodes alone can separate various cell types; however, the process takes twice as long as the design presented in this study. This extended separation time increases the risk of cell damage, even when using a voltage below the electroporation threshold. Lab-On-a-Chip devices are widely recognized for their speed advantage over traditional methods, and the presented design makes a significant difference. Choosing the presented design over a device with only 3D cylindrical electrodes would not only reduce the risk of cell damage but also improve the overall efficiency of the separation process.

Our design can separate multiple cell types from each other in one cycle of separation, and the hybrid design enables a faster separation of cells. It also increases cell viability by reducing the time cells spend when trapped by 3D electrodes. There are other devices to separate multi-type cells, for example the proposed device for cell separation by Siani et al. [55], which can separate four types of cells with a high accuracy, but it is limited to four types. Our device has no limitation in the number of bioparticle types. The only limitation of our design is the number of cells that can be trapped, but this can easily be addressed by adding more 3D cylindrical electrodes, in proportion to the sample volume. To the best of our knowledge, such a hybrid design to separate multi-types of cells has not been previously reported.

## 5. Conclusions

This study presents a novel microfluidic device design based on dielectrophoresis (DEP) for the efficient and accurate separation of multiple particle types. The hybrid design of the device incorporates two sets of electrodes, 3D cylindrical electrodes for particle trapping and 3D sidewall electrodes for particle separation, to overcome the limitations of previous particle separation methods. The results showed that the optimal electrode size and operating conditions could be determined to achieve high separation accuracy while preserving cell viability, which is crucial for post-processing analysis. The optimum operating conditions were 6 Vp-p at 35 kHz on the 3D cylindrical electrodes to trap T-cells and U937-MC cells, 20 Vp-p at 1.1 MHz on the sidewall electrodes to separate RBCs from bacteria, and 11 Vp-p at 10 kHz to separate T-cells from U937-MC cells.

The transmembrane voltage was also investigated to assure cell viability, and the results indicated that the maximum electric fields experienced by the bioparticles were below the electroporation threshold. This makes the proposed device a highly effective and reliable tool for multiparticle separation in various fields such as cell analysis, biotechnology, and medical diagnostics.

The recovery rate for each type of cell was calculated with simulations and was found to be high, with the recovery rate for RBCs, bacteria cells, T-cells, and U937-MC cells obtained as 98.2%, 95.5%, 99%, and 100%, respectively. The selectivity and separation efficiency of the proposed system were found to be approximately 99.9% and 99.8% for RBCs, 92.5% and 95.5% for bacteria cells, 100% and 98.9% for T-cells, and 95.8% and 100% for U937-MC cells, respectively.

In summary, this study presents a highly promising solution for the efficient and accurate separation of multiple particle types. Future research directions will focus on the evaluation of the device performance in real-life applications.

## Figures and Tables

**Table 1:** A summary of recent numerical studies on microfluidic devices.

Study Objective	Simulation Software	System Efficiency	Ref.
Optimization of a DEP-base microfluidic device for cancer cell separation with preserving cell viability.	COMSOL Multiphysics	95%	[30]
Numerical modeling of an ultrasonic-based system for microparticles separation.	COMSOL Multiphysics	100%	[34]
Design and numerical analysis of a novel DEP-based microparticle separator device.	COMSOL Multiphysics	Close to 100%	[35]
Optimization of a microfluidic device based on direct current dielectrophoresis to separate breast cancer cells from red and white blood cells.	COMSOL Multiphysics	100%	[36]
Numerical investigation of a novel hybrid microfluidic design for cell sorting and continuous separation of CTCs from RBCs.	COMSOL Multiphysics	97%	[31]
Numerical optimization of a multistage hybrid microfluidics platform that utilizes inertial and acoustic forces for particle separation.	COMSOL Multiphysics	-	[8]
Numerical optimization of a DEP-based cell separation device	Open FOAM, COMSOL Multiphysics	-	[32]
Modeling and optimization of a novel DEP-based multitarget cell separation microfluidic device	COMSOL Multiphysics	-	[37]
Numerical investigation and optimization of a DEP-based microfluidic platform for continuous CTC separation from blood stream	COMSOL Multiphysics	100%	[38]

**Table 2:** Geometric parameters of the proposed microfluidic device.

The Parameter	Symbol	Value
Height of microfluidic channel [µm]	h_ch_	100
Height of 3D cylindrical electrodes [µm]	h_3D_	90
Height of sidewall electrodes [µm]	h_sw_	100
Total length of the device [µm]	L_Tot_	3200
Length of the microchannel from the buffer inlets to the outlet [µm]	L_ch_	1350
Width of the sample inlet [µm]	W_I1_	540
Width of buffer inlets [µm]	W_I2_	140
Width of the microchannel in Section III [µm]	W_ch_	200
Width of side outlets [µm]	W_so_	140
Width of the outlet in the middle [µm]	W_mo_	130
Diameter of 3D cylindrical electrodes [µm]	D_3D_	50
Edge to edge horizontal distance between 3D cylindrical electrodes [µm]	d_3Dh_	100
Edge to edge vertical distance between 3D cylindrical electrodes (column 1–6 and 11) [µm]	d_3Dv1_	100
Edge to edge vertical distance between 3D cylindrical electrodes (column 7 and 9) [µm]	d_3Dv2_	170
Length of 3D sidewall electrodes [µm]	L_sw_	200
Edge to edge distance between 3D sidewall electrodes [µm]	d_sw_	100

**Table 3:** The working principle of the proposed microfluidic device.

Cell Group to Separate	Separation Route		Separation Step	Particle Trajectory		
				Group #1	Group #2	Group #n
	3D Electrode Frequency [Hz]	Sidewall Electrode Frequency [Hz]		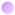 Cell #1−1 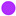 Cell #1−2	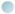 Cell #2−1 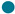 Cell #2−2	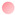 Cell #n−1 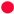 Cell #n−2
Group #1	f_3D,1_	f_sw,1_	Step I	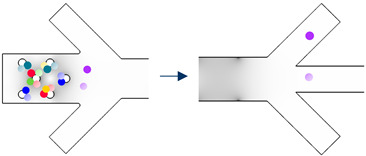		
Group #2	f_3D,2_	f_sw,2_	Step II	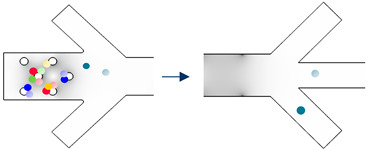		
Group #n	off	f_sw,n_	Step n	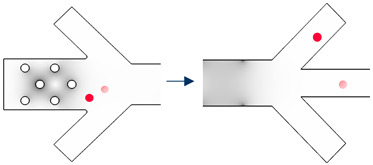		

**Table 4:** The bioparticle properties [49].

Bio Particle	Radius [mm]	Membrane Thickness [nm]	Cytoplasm Permittivity [-]	Cytoplasm Conductivity [S/m]	Membrane Permittivity [-]	Membrane Conductivity [S/m]
RBC	2.8	4.5	59	0.31	4.44	10 × 10^−6^
Bacteria	0.5	9	55	0.46	7	2.5 × 10^−5^
T-cell	3.29	7.5	103.9	0.73	8.89	2.7 × 10^−5^
U937-MC	7	7	50	0.5	12.5	1 × 10^−6^

## Data Availability

Not applicable.

## Nomenclature

r	Radius	J	Current density
CM	Clausius–Mossotti	Q	Charge density
f_CM_	Clausius–Mossotti factor	P	Pressure
Re	Real	u	Velocity vector
E	Electric field	v_p_	Particle velocity
F_DEP_	DEP force	m	mass
F_Drag_	Drag force	DEP	Dielectrophoresis
D	Diameter	AC	Alternating current
d	Thickness of the membrane	nDEP	Negative DEP
h	Height of electrodes	pDEP	Posetive DEP
L	Microchannel length	RBC	Red blood cells
W	Microchannel width	CTC	Circulating tumor cell
V_p-p_	Pick to pick voltage	d_3D_	Distance between 3D electrodes
f	Applied frequency	d_sw_	Distance between planar electrodes
t	Time		
Greek			
ε	Permittivity	𝛻	Notation for gradient
σ	Conductivity	φ	Applied potential
ω	Angular frequency	η	Dynamic viscosity
µ	Dynamic viscosity		
